# Perfusion Visualization during Ileal J-Pouch Formation—A Proposal for the Standardization of Intraoperative Imaging with Indocyanine Green Near-Infrared Fluorescence and a Postoperative Follow-Up in IBD Surgery

**DOI:** 10.3390/life12050668

**Published:** 2022-04-30

**Authors:** Leonard A. Lobbes, Susanne Berns, René Warschkow, Leonard R. Schmidt, Christian Schineis, Rahel M. Strobel, Johannes C. Lauscher, Katharina Beyer, Benjamin Weixler

**Affiliations:** 1 Department of General and Visceral Surgery, Charité—Universitätsmedizin Berlin, Corporate Member of Freie Universität Berlin and Humboldt-Universität zu Berlin, Hindenburgdamm 30, 12203 Berlin, Germany; susanne.berns@charite.de (S.B.); leonard.schmidt@charite.de (L.R.S.); christian.schineis@charite.de (C.S.); rahel.strobel@charite.de (R.M.S.); johannes.lauscher@charite.de (J.C.L.); katharina.beyer2@charite.de (K.B.); benjamin.weixler@charite.de (B.W.); 2 Department of General, Visceral, Endocrine and Transplant Surgery, Kantonsspital St. Gallen, 9000 St. Gallen, Switzerland; rene.warschkow@kssg.ch; 3 Institute of Medical Biometry and Informatics, University Heidelberg, 69120 Heidelberg, Germany

**Keywords:** ileal J-pouch, anastomotic leak, ulcerative colitis, perfusion visualization, indocyanine green (ICG) near-infrared fluorescence

## Abstract

Background: An anastomotic leak (AL) after a restorative proctocolectomy and an ileal J-pouch increases morbidity and the risk of pouch failure. Thus, a perfusion assessment during J-pouch formation is crucial. While indocyanine green near-infrared fluorescence (ICG-NIRF) has shown potential to reduce ALs, its suitability in a restorative proctocolectomy remains unclear. We aimed to develop a standardized approach for investigating ICG-NIRF and ALs in pouch surgery. Methods: Patients undergoing a restorative proctocolectomy with an ileal J-pouch for ulcerative colitis at an IBD-referral-center were included in a prospective study in which an AL within 30 postoperative days was the primary outcome. Intraoperatively, standardized perfusion visualization with ICG-NIRF was performed and video recorded for postoperative analysis at three time points. Quantitative clinical and technical variables (secondary outcome) were correlated with the primary outcome by descriptive analysis and logistic regression. A novel definition and grading of AL of the J-pouch was applied. A postoperative pouchoscopy was routinely performed to screen for AL. Results: Intraoperative ICG-NIRF-visualization and its postoperative visual analysis in 25 patients did not indicate an AL. The anastomotic site after pouch formation appeared completely fluorescent with a strong fluorescence signal (category 2) in all cases of ALs (4 of 25). Anastomotic site was not changed. ICG-NIRF visualization was reproducible and standardized. Logistic regression identified a two-stage approach vs. a three-stage approach (Odds ratio (OR) = 20.00, 95% confidence interval [CI] = 1.37–580.18, *p* = 0.029) as a risk factor for ALs. Conclusion: We present a standardized, comparable approach of ICG-NIRF visualization in pouch surgery. Our data indicate that the visual interpretation of ICG-NIRF alone may not detect ALs of the pouch in all cases—quantifiable, objective methods of interpretation may be required in the future.

## 1. Introduction

Restorative proctocolectomy (RPC) followed by the formation of an ileal pouch reservoir and ileal pouch-anal anastomosis (IPAA) as first described by Parks and Nicholls is the surgical procedure of choice for patients with inflammatory bowel disease (IBD) in cases of medically refractory ulcerative colitis (UC), UC-associated neoplasia, familial adenomatous polyposis (FAP) as well as other conditions including synchronous colorectal cancer [1,2,3,4]. Depending on the preoperative condition of the patient and the severity of disease, a two- or three-stage approach is usually chosen [5,6]. The formation of a J-shaped pouch has become the most common technique, due to its practicality, functionality and favorable long-term results [3,7]. Although the reported quality of life after RPC is good, complications such as an anastomotic leak (AL) of the pouch, pelvic sepsis, pouch necrosis, strictures, fistulas and sinuses as well as pouchitis are not uncommon [8,9,10,11,12]. As inflammatory Bowel Disease (IBD) is multifactorial from pathogenesis to manifestation to management, requiring both complex medical and surgical treatment, complications have also been shown to be multifactorial, with other important factors such as an altered immune response [13,14] having roles to play. In particular, early postoperative complications including pelvic sepsis increase the risk of pouch failure and have an adverse effect on long-term quality of life after RPC [12,15,16]. Localized septic complications have a reported incidence of 7–36% and are primarily caused by an AL of the pouch [11,16,17,18]. Restricted blood perfusion to the pouch has been reported to be a potential cause [19,20]. Hence, the assessment of blood perfusion and a tension-free anastomosis during pouch formation are critical to reduce morbidity. To date, bowel perfusion is intraoperatively assessed by parameters such as the color of bowel serosa or mucosa, the amount of bleeding from transection sites and the palpability of a pulse on vascular arcades. These criteria are highly subjective, depending on individual experience and the degree of specialization of the surgeon and lack the sensitivity and specificity for ALs [21,22].

Indocyanine green derived near-infrared fluorescence (ICG-NIRF) enables the clinician to visualize bowel perfusion in real-time [23,24]. The technique has shown potential to reduce the incidence of ALs after laparoscopic colorectal resections, improving the outcome of bowel anastomoses in terms of safety and efficacy [23,25]. However, despite the supposed objectivity of ICG-NIRF, its clinical interpretation is still largely subjective. This is because important physiological and technical parameters relevant for visualization at the time of ICG administration (e.g., mean arterial blood pressure, the use of vasoactive drugs, the distance between the camera and the imaging site) are barely considered in the work published and studies on ICG-NIRF therefore lack standardization. Data on the suitability of ICG-NIRF for perfusion visualization in pouch surgery are scarce, as are objective, standardized trials to assess its suitability for anastomotic evaluation in general.

This study aimed to evaluate ICG-NIRF for the intraoperative detection of ischemia-related complications in ileal J-pouch formation and to develop a standardized approach for an ICG-NIRF perfusion assessment to improve data comparability of future trials.

## 2. Materials and Methods

This prospective cohort study (NCT04695184) was performed at the Department of General and Visceral Surgery, Charité University Hospital Berlin, Campus Benjamin Franklin, a tertiary colorectal and inflammatory bowel disease (IBD) referral center, from February 2019 to December 2020. The study protocol was approved by the Charité University Hospital Ethics Committee (EA4/116/19, Ethikkommission der Charité—Universititätsmedizin Berlin) and conducted in agreement with the Declaration of Helsinki. Data were reported conforming to the STROBE guidelines [26]. 

Patients undergoing ileal J-pouch formation either in a two- or three-stage approach were eligible for study inclusion. In the two-stage approach, patients underwent a proctocolectomy simultaneously with ileal J-pouch formation and a loop ileostomy. Patients undergoing the three-stage approach received a completion proctectomy with ileal J-pouch formation and a loop ileostomy following a prior subtotal colectomy with an end ileostomy. Inclusion criteria were a diagnosis of medically refractory ulcerative colitis, indeterminate colitis, colorectal cancer, FAP, age ≥ 18 years and an American Society of Anesthesiologists (ASA) physical status ≤ 3, laparoscopic and open surgery. Exclusion criteria were the coexisting malignancy of a different etiology, liver dysfunction (MELD score > 10), hypersensitivity to indocyanine green (ICG) or sodium iodide, iodine allergy, hyperthyroidism, thyroid nodules, a previously poorly tolerated injection of ICG, as well as pregnancy and breastfeeding.

Written informed consent for participation and ICG-administration was obtained on the day before surgery. 

### 2.1. Surgical Technique

Following the standard procedure of a high-volume tertiary IBD referral center, ileal pouch-anal anastomosis (IPAA) was standardly achieved without a mucosectomy by circular mechanical double-stapling using a 29 mm end-to-end anastomosis (EEA) stapler after fashioning the J-pouch to a length of 14–16 cm by linear single-stapling (and ensuring tension-free IPAA by checking that the mobility of the pouch reached down to the symphysis pubis extracorporeally). In cases where this technique was not possible following good clinical practice due to the risk of malignancy, single-layer hand-sewn anastomosis was performed after the completed mucosectomy.

### 2.2. Intraoperative NIRF Imaging

Intraoperatively, ICG (VerDye, Diagnostic Green GmbH, Aschheim Germany, 25 mg vials) was dissolved in 5 mL sterile water to yield a 5 mg/mL concentration and administered intravenously as a bolus of 1 mL (5 mg), respectively, at three consecutive time points. Real-time intraoperative perfusion visualization was performed with the Quest Spectrum^®^ Fluorescence Imaging Platform (Quest Medical Imaging, Middenmeer, The Netherlands) directly after each ICG injection. 

**Figure 1:**
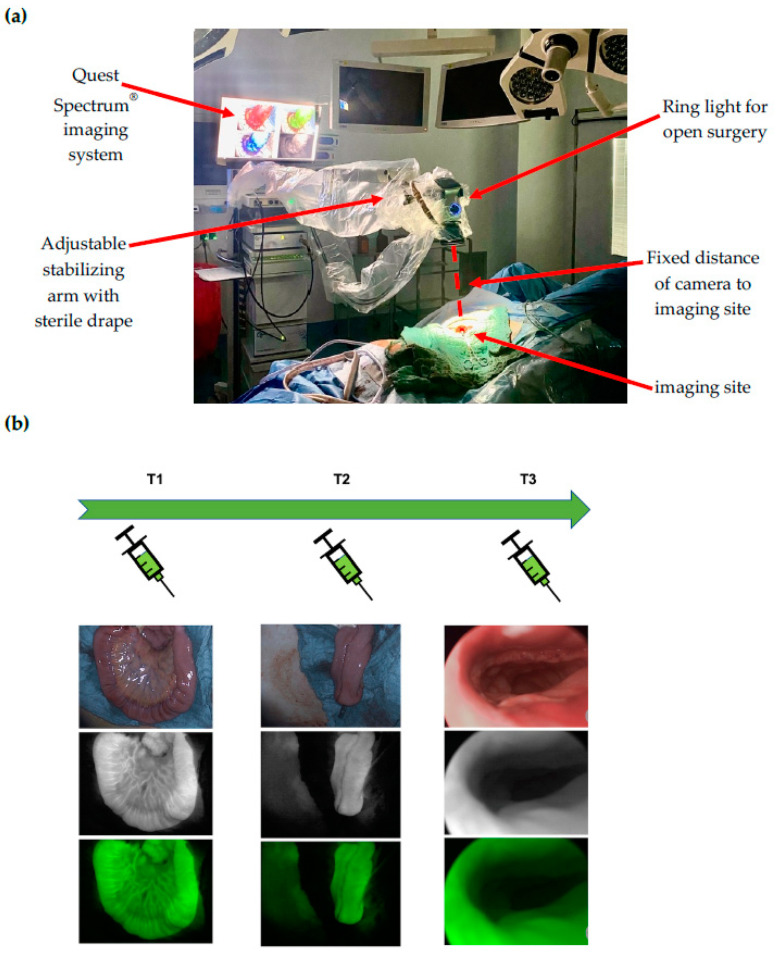
(**a**) Intraoperative set-up of the Quest Spectrum imaging system: At time points 1 and 2, the Quest Spectrum^®^ system was equipped with the ring light camera for open surgery, which was adjusted manually in position and focused for the optimal visualization of the imaging site and secured in position by an attachment to the stabilizing arm to ensure a fixed distance to the imaging site; (**b**) Time points and ICG-NIRF visualization: At time point 1 (T1), the terminal ileal loop was visualized prior to J-pouch formation. At time point 2 (T2), the J-pouch was visualized after formation by side-to-side stapled anastomosis. At time point 3 (T3), the ileal pouch-anal anastomosis (IPAA) was visualized after completion by trans-anal circular stapling. For each time point, the white light (top image), fluorescence (middle image) and overlay visualization (bottom image) is depicted.

At time point 1 (T1), the terminal ileal loop was visualized prior to J-pouch formation. At time point 2 (T2), the J-pouch was visualized immediately after formation by side-to-side stapled anastomosis. At time point 3 (T3), the IPAA was visualized after completion by trans-anal circular stapling. At time points 1 and 2, the Quest Spectrum^®^ system was equipped with the ring light camera for open surgery, which was adjusted manually in position and focused for the optimal visualization of the imaging site and secured in position by an attachment to the stabilizing arm to ensure a fixed distance to the imaging site (Figure 1a). At time point 3, the Quest Spectrum^®^ laparoscope was inserted anally through a 12 mm laparoscopy port (Versaport™ Bladeless Optical Trocar, Medtronic, Minneapolis, MN, USA), to protect the IPAA while allowing 360° visualization by manual air insufflation (Figure 1b).

At each time point, the time period from ICG injection to the visual detection of a full fluorescent signal was video recorded for postoperative analysis in the fluorescent mode and the overlay mode of fluorescence and color image (time to fluorescence signal/time to overlay signal). Additional clinical and technical data were documented, as well as the timing between the subsequent measurements. It was noted whether the operating surgeon decided to change the area of transection according to the ICG-NIRF signal. 

Postoperatively, the video recordings of visualization were analyzed to determine the quantitative subjective parameters. The clinical outcome was unknown to the investigator at this point. Fluorescence signal strength was categorized as “0”, i.e., signal not detectable; “1”, i.e., signal detectable; or “2”, i.e., strong signal detectable. It was further categorized as “homogenous”, i.e., uniformly distributed across the bowel included in the imaging site; or “heterogeneous”, i.e., unevenly distributed across the bowel included in the imaging site. Complete fluorescence of the pouch anastomotic site was categorized at time points T2 and T3 as “Yes”, i.e., completely fluorescent; or “No”, i.e., not completely fluorescent. 

An AL of the pouch was defined as the primary outcome. A change of the anastomotic site was not intended based on NIRF visualization. The secondary outcome consisted of clinical and technical data during and after visualization.

### 2.3. Clinical Data and Follow-Up

Clinical data were collected from all patients on 30-day postoperative morbidity, mortality and length of hospital stay, including ALs of the pouch, pouch necrosis and pouchitis. An endoscopy (pouchoscopy) with a flexible endoscope was performed six to eight weeks after hospital discharge or during the postoperative hospital stay if signs of ALs or pelvic sepsis (leukocytosis, elevated C-reactive protein (CRP), fever > 38.5 °C, prolonged postoperative ileus) were detected.

### 2.4. Anastomotic Leak of the Pouch—Definition and Grading

To date, there is no standardized definition of an AL of the ileal pouch. Thus, in conformity with previous definitions of an anastomotic leak in colorectal surgery [27], we specified and applied the following definition: an anastomotic leak of the pouch is defined as a defect of intestinal wall integrity at the anastomotic sites of the pouch leading to a communication between intra- and extraluminal compartments and an exodus of pouch luminal content (including air, fecal content and abscess formation communicating with the anastomotic site). Diagnosis is made by pouchoscopy and can be supported radiographically (CT or MRI). It can occur with or without the clinical presentation of symptoms related to pelvic sepsis. In accordance with the grading of anastomotic leaks after resection of the rectum [27], we further defined the following clinical grading of an anastomotic leak of the pouch:

Grade A: Anastomotic leak of the pouch requiring no active therapeutic intervention.

Grade B: Anastomotic leak of the pouch requiring active therapeutic intervention, but manageable without a relaparotomy.

Grade C: Anastomotic leak of the pouch requiring a relaparotomy. 

### 2.5. Statistical Analysis

Statistical analyses were performed using R statistical software (version number 4.1.2, www.r-project.org, the R foundation, Vienna, Austria; accessed on 1 December 2021). A two-sided *p* value < 0.05 was considered statistically significant. Continuous data are expressed as the means (standard deviation) or medians with interquartile ranges (IQRs). Proportions were compared by Mid-P-chi-square statistics or Monte Carlo (MC) simulated chi-square statistics as appropriate. Continuous variables were compared by Mann–Whitney U-tests. Leakage was further assessed by logistic regression analysis. *p* values were estimated by likelihood-ratio tests. For continuous predictors, the *p* values were estimated based on the mean ranks [28]. Coefficients and their 95% confidence intervals (CI) were estimated by the Wald method. 

## 3. Results

**Figure 2:**
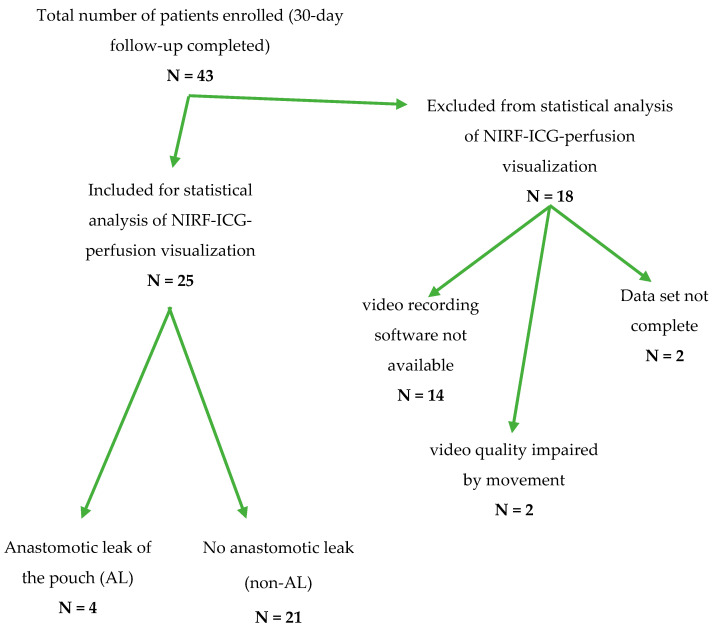
Flow-chart depicting the transition of the total number of patients recruited from February 2019 to December 2020 to the number of patients included in the statistical analysis of ICG-NIRF data regarding an anastomotic leak of the ileal J-pouch after exclusion for the respective reasons.

Overall, 43 patients undergoing ileal pouch surgery consented for study inclusion (Table A1). To ensure a valid and accurate correlation of the clinical and technical parameters of ICG-NIRF visualization with patient outcomes and the occurrence of ALs, a complete data set including a video recording and complete documentation were required. At the beginning of patient recruitment, a software solution for recording ICG-NIRF visualization was not available. To keep a reliable, high standard of reproducibility, 18 patients were excluded from the statistical analysis (Figure 2). A detailed clinical follow-up was nonetheless conducted for all 43 patients (Table A1). 

A total of 25 patients were included for the statistical analysis of which 13 were female, 24 had the diagnosis of UC and one had the diagnosis of IC (Table 1). 

Twenty patients underwent laparoscopically assisted surgery, and five patients underwent open surgery. A total of 23 patients received stapled IPAA and 2 patients received handsewn anastomosis for oncologic reasons. Twenty-two patients underwent a three-stage approach, while three patients underwent a two-stage approach. The median age at operation was 34.8 years (inter quartile range [IQR] 31.1 to 45.8). No mortality occurred. No intraoperative changes of the transection or anastomotic site took place either due to conventional clinical judgement or ICG-NIRF findings. Tension-free anastomosis was achieved in all patients. There was no use of vasoactive agents such as norepinephrine during visualization. An intraoperative erythrocyte transfusion was administered in only one patient (AL). 

The primary outcome anastomotic leak of the pouch (AL) was detected in 4 of 25 patients, all of which were classified as grade B. Statistical analysis indicated that there were no significant differences in patient characteristics and intraoperative surgical data when comparing the AL-group to the non-AL group (Table 1).

Out of the total collective of 43 patients, 6 patients had ALs, 1 of which was graded as A, 4 were graded as B and 1 was graded as C. Postoperative complications occurred in 18 patients and were listed in detail and categorized using the Clavien–Dindo classification of surgical complications (Table A1) [29]. 

Additionally, detailed clinical and technical parameters at the time points of ICG-NIRF visualization T1–T3 were determined for statistical analysis (Table 2). The administered dose of ICG per time point was consistently 5 mg. Mean arterial blood pressure (MAP), heart rate (HR) and peripheral oxygen saturation (SpO2) did not diverge significantly (Table 2). The anastomotic site appeared completely fluorescent in every patient, including the AL cohort. Subjective signal strength was categorized as 2 (strong signal) and homogenous in the majority of cases and all cases with ALs. The mean time from the injection of ICG to the visual detection of a fluorescence signal (time to fluorescence signal) was 17 s at T1 and 18.3 s at T2. The mean time period between visualizations T1 and T2 was 19.5 min and in 40% of cases, the fluorescence signal was still detectable before the second injection of ICG. The mean distance of the camera to the imaging site was 36.6 cm at T1 and 37.2 cm at T2. No significant difference was found with regards to the occurrence of an AL of the pouch when comparing the AL-group to the non-AL group. 

In univariable logistic regression analysis, only the two-stage approach (Odds ratio (OR) = 20.00, 95% confidence interval [CI] = 1.37–580.18, *p* = 0.029) was identified as a significant risk factor for ALs (Table 3).

Univariable logistic regression of technical parameters at the time of ICG-NIRF visualization was performed additionally and showed no statistical difference for the event of an AL of the pouch.

## 4. Discussion

As intraoperative ICG-derived near-infrared fluorescence imaging is becoming increasingly popular, publications in this field are accumulating fast. While the potential of the technique has been shown, its validity and reproducibility remain unclear, as there are considerable differences in its individual application and interpretation. Thus, establishing and maintaining a high scientific standard when conducting ICG-NIRF studies is acutely important. In the work presented here, we focused on an accurate and reproducible methodology, aiming for a comparability of data with other and future studies. In particular, our methodology addressed variables for enabling a quantitative comparison of ICG-NIRF visualization data. In the present study, ICG-NIRF was conducted in a standardized and controlled manner, including the distance and timing of visualization. Quantitative subjective criteria of fluorescence were defined and applied in the postoperative blinded analysis. The results demonstrated that ICG-NIRF was reproducible, did not indicate an AL of the pouch within 30 days post-operation and identified a two-step surgical approach as a significant risk factor for AL (Table 3). Three out of four patients with an AL received additional immunosuppressive medication (Table 1). There were no non-IBD patients included due to the nature of the surgical indication for the procedure investigated. This makes the results representative and relevant to IBD specialists but may add complexity to the underlying mechanisms behind complications.

In several recent reviews on fluorescence-guided abdominal surgery, a lack of quantitative, unbiased data in the available literature and the need for further research into the specifics of its application were pointed out [24,30,31,32]. The authors of these reviews remarked that the reported approaches of vascular perfusion assessment with ICG do not take into account the accumulation of ICG over time. To date, the visual interpretation of ICG-NIRF results in areas that appear fluorescent and areas that do not, regardless of the timing of ICG injection and visualization. While this might be sufficient to indicate areas with an absolute lack of perfusion in terms of arterial inflow of blood, it may not be able to detect locally decreased microperfusion and venous congestion in the case of an AL, as our results suggest. This may lead to an overestimation of the fluorescence in the tissue of interest. Waiting for the elimination (“wash-out”) of ICG from the tissue of interest would not be practicable, as our results showed that the signal was still detectable after 18–20 min. These are factors that need to be considered for the assessment of bowel perfusion. The literature confirms this: although the changes in the anastomotic site due to a weak overall ICG signal have occasionally been described, the characteristics of a relative perfusion deficit or an AL have not been shown in visual detection alone, even in larger, randomized controlled trials [33].

As the comparison of time points T1, T2 and T3 shows, the methodology presented here is quantitative, accurate and reproducible, allowing a comparison of subsequent ICG-NIRF visualizations and takes into account previously unreported variables. It seems probable that the time points T1 (before pouch formation), T2 (after pouch formation) and T3 (after completed IPAA) have different degrees of relevance for assessing postoperative pouch perfusion. T1 was chosen to indicate the baseline perfusion of the ileum segment to be used for ileal pouch formation in order to investigate reproducibility and potentially detect areas of initially impaired perfusion after bowel mobilization and transection. The visualization of the J-pouch at T2 after its formation has a conceivably high validity in assessing postoperative pouch perfusion, although perfusion may additionally be affected after delivery of the pouch down into the pelvis. The visualization at T3 can potentially indicate perfusion across the IPAA, which can be regarded as the Achilles’ heel of the ileoanal pouch. However, at T3, the technical difficulty of standardizing the endoluminal field of view in a dynamic environment may have affected the validity of the visualization. Advances in the form of near-infrared fluorescence endoscopes are needed, as this imaging location and timing is vital for assessing the perfusion of ileoanal and colorectal anastomoses.

Despite the standardized quantitative analysis of ICG-NIRF visualization, no significant differences were seen in patients developing an AL of the pouch. The intraoperative fluorescence signal was completely fluorescent at the anastomotic site in all patients with an AL. This was reproduced in the postoperative analysis of the video recordings and demonstrates that the visual interpretation of ICG-NIRF remains subjective irrespective of quantification and standardization. Subjective ICG-NIRF visualization does not seem to be sufficient to completely assess pouch microperfusion to detect an AL. Anastomotic tension could have contributed to the occurrence of an AL; however, tension-free anastomosis was achieved in all patients, excluding this factor as a cause. 

While numerous studies have adequately described the feasibility of ICG-NIRF for visualizing overall perfusion before or after intestinal anastomosis [23,32,34,35,36], data addressing ileal J-pouch perfusion and the AL of the pouch are scarce. To date, one case report and one case-matched study reported using ICG-NIRF in assessing ileal pouch perfusion [37,38]. In the latter, Spinelli et al. visualized pouch perfusion at similar time points. However, three different camera systems were used, which raises the question of inter-device comparability, as imaging devices may differ in the degree of fluorescence visualization and signal enhancement. There was no quantitative description or interpretation of the intraoperative visualization nor video recordings to ensure reproducibility, which raises the question of how a sufficient fluorescence signal was defined. As a pouchoscopy was not part of the follow-up, it is unclear how an AL of the pouch was identified, especially in cases of asymptomatic ALs due to an ileostomy. The cohort selected for case-matched historical comparison contained only one case of an AL, which suggests that a low-risk cohort of patients was included for ICG-NIRF assessment. 

In our study, a pouchoscopy was included in the follow-up of each patient, which explains the higher rate of AL detection. Thus, we identified ALs of the pouch in 4 out of 25 (16%) patients. In the literature, the reported rates of ALs of the pouch differ considerably, most often ranging from 6 to 10%. However, complications such as pelvic sepsis range from 7 to 36% and may likely occur due to an AL of the pouch. Additionally, to date there is no standardized definition or grading for an AL of the pouch, which suggests that there are significant differences in its detection. A considerable occurrence of asymptomatic ALs of the pouch masked by the presence of an ileostomy has already been described in the literature [39,40]. In a previous meta-analysis, only a limited number of included studies assessed the J-pouch, despite it being the current gold-standard in restorative proctocolectomy [41]. In other meta-analyses, pelvic sepsis and pouch failure have been reported, while the actual rate of AL of the pouch was not [42]. Thus, the question of the actual rate of ALs of the pouch remains unanswered. Recent studies have aimed to improve the definition of an AL of the pouch [43]. In our data set, we applied a precise definition and methodology, which suggests that the (higher) rate of ALs in our data is closer to reality. In reference to the grading of an AL after the resection of the rectum, we used a definition and grading of an AL of the pouch, which we propose for future studies on ALs in RPC. 

Finally, we acknowledge the limitations of the presented study. We conducted a study with a limited number of patients without randomization. Therefore, the impact and reliability of the statistical analysis may be limited. Accrual was mainly limited due to the later availability of a software allowing the video recording of NIRF visualization for a postoperative, strictly accurate assessment and the high degree of standardization and detail during visualization, requiring extensive data sets. However, we aimed to control for an extensive number of variables during ICG-NIRF visualization, therefore reducing potential confounders to a minimum. While the descriptive analysis of patient characteristics and the technical data of ICG-NIRF visualization did not provide significant differences for patients with an AL of the pouch, logistic regression did identify a two-stage approach as a risk factor for ALs, which has been repeatedly reported previously [2,5,6]. This indicates that the data collected were robust. SpO2 at T3 was close to statistical significance for ALs, the reason for which should be investigated in larger trials with numerical power. One possibility is reduced oxygen perfusion to the anastomotic site in the case of overall or bordering hypoxia.

Despite the relatively small sample size and thus possibly a limited external validity, the findings may indicate that a visual interpretation of ICG-NIRF visualization may not be objective or detailed enough to indicate cases of impaired microperfusion leading to an AL. Follow-up studies including objective interpretation methods and an evaluation of different techniques are needed. Additionally, other surgery- and patient-related factors contributing to ALs will continue to be of investigative importance to improve patient outcomes.

## 5. Conclusions

The findings of this study provide a methodology for standardizing ICG-NIRF perfusion visualization and provide a model for investigating the outcome of ileal pouch surgery. They may further indicate that the visual interpretation of ICG-NIRF alone may not be sufficient to assess the risk of an AL of the J-pouch in RPC intraoperatively. Objective methods of interpretation may be required for valid intraoperative perfusion assessments.

## Figures and Tables

**Table 1:** Patient characteristics and intraoperative surgical data.

Characteristic	All Patients (N = 25)	AL (n = 4)	Non-AL (n = 21)	*p*-Value
**Sex (n)**				0.937 (A)
**female**	13	2	11	
**male**	12	2	10	
**Age at operation (years) mean** **(SD)** **range**	36.5 (±10.5) 19.9–57.0	40.7 (±10.2) 34.8–56.0	35.7 (±10.6) 19,9–57.0	0.282 (B)
**BMI (kg/m^2^) mean** **(SD)** **range**	24.2 (±5.0) 18.2–35.3	24.7 (±6.9) 18.2–33.1	24.1 (±4.7) 18.6–35.3	0.803 (B)
**ASA (Class)**				1.000 (C)
**I**	2	0	2	
**II**	20	3	17	
**III**	3	1	2	
**Underlying condition (n)**				0.160 (A)
**IC**	1	1	0	
**UC**	24	3	21	
**Disease category (n)**				0.700 (A)
**malignancy**	2	0	2	
**medically refractory**	23	4	19	
**Preoperative prednisolone**				0.840 (A)
**Yes**	1	0	1	
**no**	24	4	20	
**Additional immunosuppressive medication**				0.210 (C)
**Yes**	7	3	4	
**No**	17	1	16	
**No information**	1	0	1	
**Surgical approach**				0.058 (A)
**Three-stage**	22	2	20	
**Two-stage**	3	2	1	
**Type of surgery**				0.783 (A)
**Laparoscopically**	20	3	17	
**Open**	5	1	4	
**IPAA technique**				0.700 (A)
**Handsewn**	2	0	2	
**Stapled**	23	4	19	
**Change of transection site**				-
**yes**	0	0	0	
**no**	25	4	21	
**Intraoperative erythrocyte transfusion**				0.840 (A)
**Yes**	1	0	1	
**no**	24	4	20	

Statistical Tests applied: (A) = mid-P exact test, (B) = Mann–Whitney U test, (C) = Monte Carlo simulated Chi-squared test; Abbreviations: n = number; AL = anastomotic leak; SD = standard deviation; BMI = body mass index; kg = kilograms; m^2^ = square meters; ASA = American Society of Anesthesiologists (ASA) physical status classification; UC = ulcerative colitis; IC = indeterminate colitis.

**Table 2:** Clinical and technical parameters at the time of ICG-NIRF visualization (time points T1–T3).

Time Point	T1				T2				T3			
	All Patients (n = 25)	Non-AL (n = 21)	AL (n = 4)	*p*-Value	All Patients (n = 25)	Non-AL (n = 21)	AL (n = 4)	*p*-Value	All Patients (n = 25)	Non-AL (n = 21)	AL (n = 4)	*p*-Value
**ICG dose (mg)** mean (SD) range	5.0 (±0) 5.0–5.0	5.0 (±0) 5.0–5.0	5.0 (±0) 5.0–5.0	-	5.0 (±0) 5.0–5.0	5.0 (±0) 5.0–5.0	5.0 (±0) 5.0–5.0	-	5.0 (±0) 5.0–5.0	5.0 (±0) 5.0–5.0	5.0 (±0) 5.0–5.0	-
**MAP (mmHg)** mean (SD) range	73.6 (±10) 63.0–97.0	74.0 (±10.6) 63.0–97.0	71.5 (±5.8) 65.0–79.0	1.000 (B)	72.7 (±9.0), 60.0–95.0	73.2 (±8.7), 60.0–95.0	70.0 (±11.3), 62.0–86.0	0.392 (B)	71.9 (±10.7) 60.0–95.0	72.9 (±11.2) 60.0–95.0	67.0 (±7.2) 61.0–75.0	0.569 (B)
**HR (min)** mean (SD) range	76.6 (±13.4) 50.0–105.0	76.7 (±14.1) 50.0–105.0	76.0 (±10.5) 68.0–90.0	0.911 (B)	74.5 (±12.5), 51.0–105.0	74.6 (±13.4), 51.0–105.0	74.0 (±7.8), 65.0–81.0	0.824 (B)	77.4 (±14.2) 49.0–110.0	74.8 (±12.6) 49.0–100.0	89.3 (17.9) 78.0–110.0	0.128 (B)
**SpO2 (%)** mean (SD) range	99.5 (±0.8) 97.0–100.0	99.6 (±0.8) 97.0–100.0	99.2 (±1.0) 98.0–100.0	0.358 (B)	99.5 (±0.9), 97.0–100.0	99.5 (±0.9), 97.0–100.0	99.2 (±1.0), 98.0–100.0	0.411 (B)	99.0 (±2.3) 93.0–100.0	99.4 (±1.9) 93.0–100.0	97.0 (±3.6) 93.0–100.0	0.062 (B)
**time to fluorescence signal (s)** mean (SD) range	17.0 (±9.7) 4.9–38.9	16.8 (±9.8) 4.9–38.9	17.8 (±10.9) 5.0–27.0	0.858 (B)	18.3 (±10.3) 1.6–42.3	18.5 (±10.1) 5.2–42.3	17.2 (±13.0) 1.6–28.8	0.795 (B)	-	-	-	-
**time to overlay signal (s)** mean (SD) range	18.7 (±10.3) 5.1–43.6	18.6 (±10.4) 5.1–43.6	19.0 (±10.7) 6.5–28.5	0.971 (B)	19.1 (±10.4), 1.6–42.6	19.3 (±10.1), 6.3–42.6	17.9 (±13.6), 1.6–29.2)	0.803 (B)	-	-	-	-
**time** **difference** **of fluorescence signal and green overlay (s)** mean (SD) range	1.7 (±1.4) 0.2–5.1	1.8 (±1.5) 0.2–5.1	1.1 (±0.5) 0.6–1.5	0.642 (B)	0.8 (±0.7) 0.0–2.5	0.8 (±0.6) 0.0–2.5	0.7 (±0.9) 0.0–2.0	0.683 (B)	-	-	-	-
**signal strength**												
0	0	0	0	0.473 (A)	0	0	0	0.840 (A)	-	-	-	0.806 (A)
1	4	4	0		1	1	1					
2	21	17	4		24	20	4		17	14	3	
ambiguous									8	7	1	
**Signal heterogeneity**												
homogenous	19	17	2	0.265 (A)	24	20	4	0.840 (A)	17	14	3	0.806 (A)
heterogenous	6	4	2		1	1	0		8	7	1	
**distance camera to site (cm)** mean (SD) range	36.6 (±8.0) 19.0- 49.0	36.7 (±8.7) 19.0–49.0	36.5 (±3.1) 33.0–40.0	0.737 (B)	37.2 (±7.2) 21.0–49.0	37.0 (±7.9) 21.0- 49.0	38.0 (±2.4) 35.0–40.0	1.0 (B)	-	-	-	-

**Statistical Tests applied**: (A) = mid-P exact test, (B) = Mann–Whitney U test; **Abbreviations:**; T1 = time point 1; T2 = time point 2; T3 = time point 3; N = number; AL = anastomotic leak; SD = standard deviation; (-) = not applicable; MAP = mean arterial blood pressure; mmHg = millimeters of mercury; HR = heart rate; min = minute; SpO2 = peripheral oxygen saturation determined by pulse oximetry; s = seconds; signal strength: of fluorescence signal: 0 = no signal, 1 = detectable signal, 2 = strong signal.

**Table 3:** Univariable logistic regression of clinical data (baseline patient data) to estimate a 95% confidence interval and Odds ratios for the event of ALs.

Characteristic	Odds Ratio (OR)	95% Confidence Interval	*p*-Value
**Sex** (male vs. female)	1.10	0.11–10.63	0.930
**age at operation** (years)	1.05	0.94–1.16	0.248
**BMI** (kg/m^2^)	1.02	0.80–1.26	0.762
**ASA** (3 vs. 1 and 2)	3.17	0.12–45.88	0.424
**Surgical approach** **(two-stage vs. three-stage)**	20.00	1.37–580.18	0.029
**Type of surgery** **(open vs. laparoscopically)**	1.42	0.06–15.08	0.790

**Abbreviations:** BMI = Body Mass Index; kg = kilograms; m^2^ = square meters; ASA = American Society of Anesthesiologists (ASA) physical status classification.

## Data Availability

Not applicable.

## Appendix A

**Table A1:** Patient characteristics and intraoperative surgical data for all patients recruited (43 patients).

Characteristic	All Patients (N = 43)	AL (n = 6)	Non-AL (n = 37)
**Sex (n)**			
**female**	18	2	16
**male**	25	4	21
**Age at operation (years) mean** **(SD)** **range**	37.7 (±11.0) 18.4–64.8		
**BMI (kg/m^2^) mean** **(SD)** **range**	24.7 (±4.6) 18.2–35.4		
**ASA (Class)**			
**I**	3	0	3
**II**	33	5	28
**III**	7	1	6
**Underlying condition (n)**			
**IC**	1	1	0
**UC**	42	5	37
**Disease category (n)**			
**malignancy**	5	0	5
**medically refractory**	38	6	32
**Preoperative prednisolone**			
**Yes**	3	0	3
**no**	40	6	34
**Additional immunosuppressive medication**			
**Yes**	9	3	6
**No**	32	2	30
**No information**	2	1	1
**Surgical approach**			
**Three-stage**	36	3	33
**Two-stage**	7	3	4
**Type of surgery**			
**Laparoscopically**	36	4	32
**Open**	7	2	5
**IPAA technique**			
**Handsewn**	6	0	6
**Stapled**	37	6	31
**Change of transection site**			
**yes**	0	0	0
**no**	43	6	37
**Intraoperative erythrocyte transfusion**			
**Yes**	1	0	1
**no**	42	6	36

**Abbreviations:** n = number; AL = anastomotic leak; SD = standard deviation; BMI = body mass index; kg = kilograms; m^2^ = square meters; ASA = American Society of Anesthesiologists (ASA) physical status classification; UC = ulcerative colitis.
